# Supplementary material for: Wolbachia and its pWCP plasmid show differential dynamics during the development of Culex mosquitoes
Source: Microbiol Spectr. 2025 Mar 31;13(5):e00046-25. doi: 10.1128/spectrum.00046-25 (PMC12054023; doi:10.1128/spectrum.00046-25)
Supplement: Data S1 — R code. [file spectrum.00046-25-s0001.pdf]

Analysis of Wolbachia and Plasmid Dynamics in Culex Mosquitoes

2024-12-19

Introduction

This R script analyzes two datasets corresponding to two distinct mosquito species, Culex pipiens molestus and Culex quinquefasciatus. The primary goals are to study the dynamics of the plasmid pWCP (GP11) and Wolbachia (wsp gene) across developmental stages, using GLM analyses and visualizations. Please note that the datasets are structured similarly, and the code applies identically to both, with only the file paths changing

```r
# Loading Required Packages

library(tidyverse)
library(readxl)
library(emmeans)
library(ggplot2)
library(performance)

# Loading the Data

raw_data_mol <-read_excel('/Users/alice/Dropbox/raw_data_pipiens_molestus.xlsx')
raw_data_quinque <-read_excel('/Users/alice/Dropbox/raw_data_quiquefasciatus.xlsx')
combined_data <-read_excel('/Users/alice/Dropbox/combined_data.xlsx')
```

GLM Analysis: wsp/ACE2

GLM Analysis and Model Check

```r
# GLM analysis for wsp/ACE2
mod1=glm(wsp_ACE2~Stade, data=raw_data_mol)
summary(mod1)

# Marginal means and pairwise comparisons
emmeans_mod1 <- emmeans(mod1, ~ Stade)

pairwise_comparisons <- pairs(emmeans_mod1, adjust = "bonferroni")

summary(pairwise_comparisons)

# Model diagnostics
check_model(mod1)
```

Visualization: wsp/ACE2

```r
# Reorganize developmental stages
raw_data_mol$Stade <- factor(raw_data_mol$Stade, levels = c("L1", "L2", "L3", "L4", "Pupa", "Female", "Male"))

# Plot wsp/ACE2 dynamics
ggplot(raw_data_mol, aes(x = Stade, y = wsp_ACE2, fill = Stade)) +
  geom_boxplot() +
  geom_jitter(position = position_jitter(0.2), size = 2, alpha = 0.6, color = "black") +
  theme_classic() +
  labs(title = "Quantity of Wolbachia depending of the stage of development",
       x = "Stage of development",
       y = "Wolbachia/Mosquitoes cells (wsp/ACE2)") +
  theme(legend.position = "none")+
  ylim(0, 8)
```

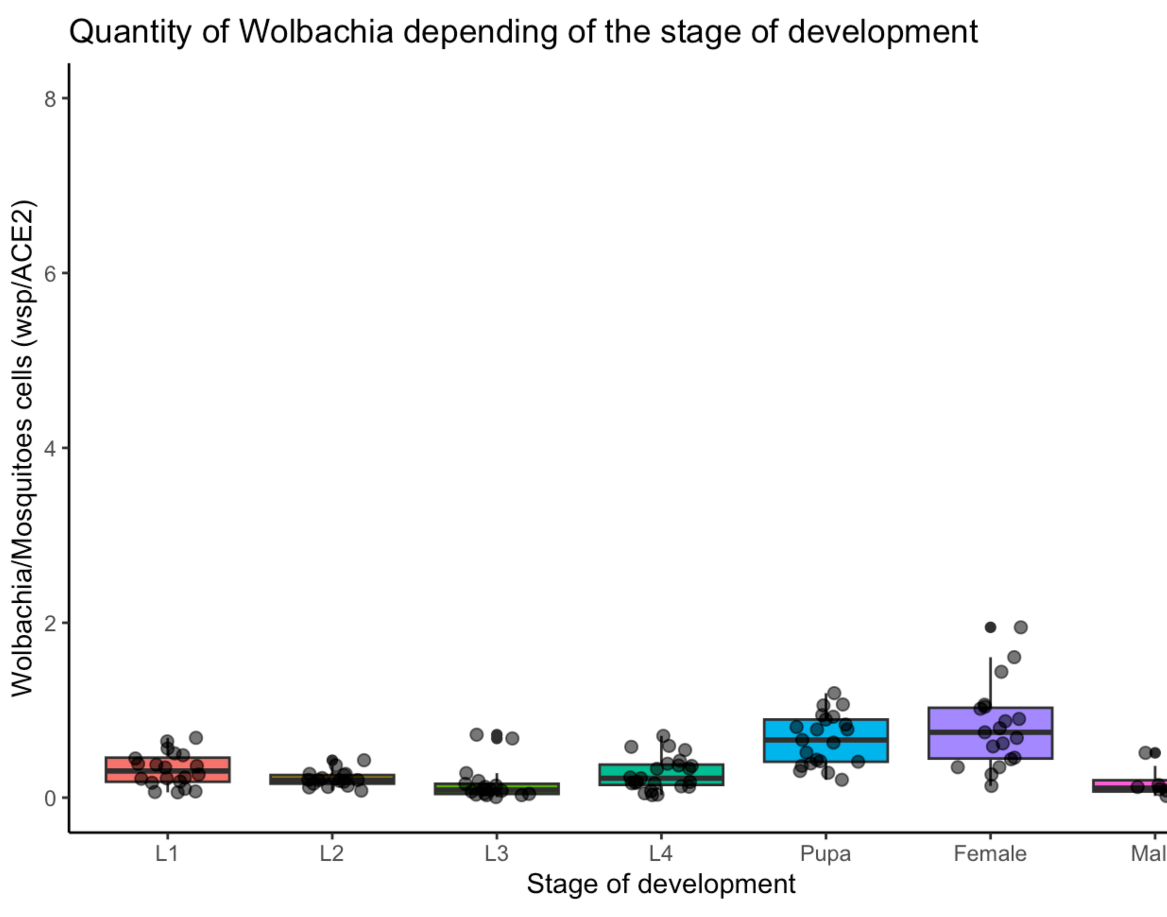

GLM Analysis: GP11/wsp

GLM Analysis and Model Check

```r
# GLM analysis for GP11/wsp
mod2=glm(GP11_wsp~Stade, data=raw_data_mol)
summary(mod2)

# Marginal means and pairwise comparisons
emmeans_mod2 <- emmeans(mod2, ~ Stade)

pairwise_comparisons_2 <- pairs(emmeans_mod2, adjust = "bonferroni")
summary(pairwise_comparisons_2)

# Model diagnostics

check_model(mod2)
```

###Visualization: GP11/wsp

```r
# Plot GP11/wsp dynamics

ggplot(raw_data_mol, aes(x = Stade, y = GP11_wsp, fill = Stade)) +
  geom_boxplot() +
  geom_jitter(position = position_jitter(0.2), size = 2, alpha = 0.6, color = "black") +
  theme_classic() +
  labs(title = "Quantity of plasmid depending of the stage of development",
       x = "Stage of development",
       y = "Plasmid/Wolbachia (GP11/wsp)") +
  theme(legend.position = "none")+
  ylim(0, 10)
```

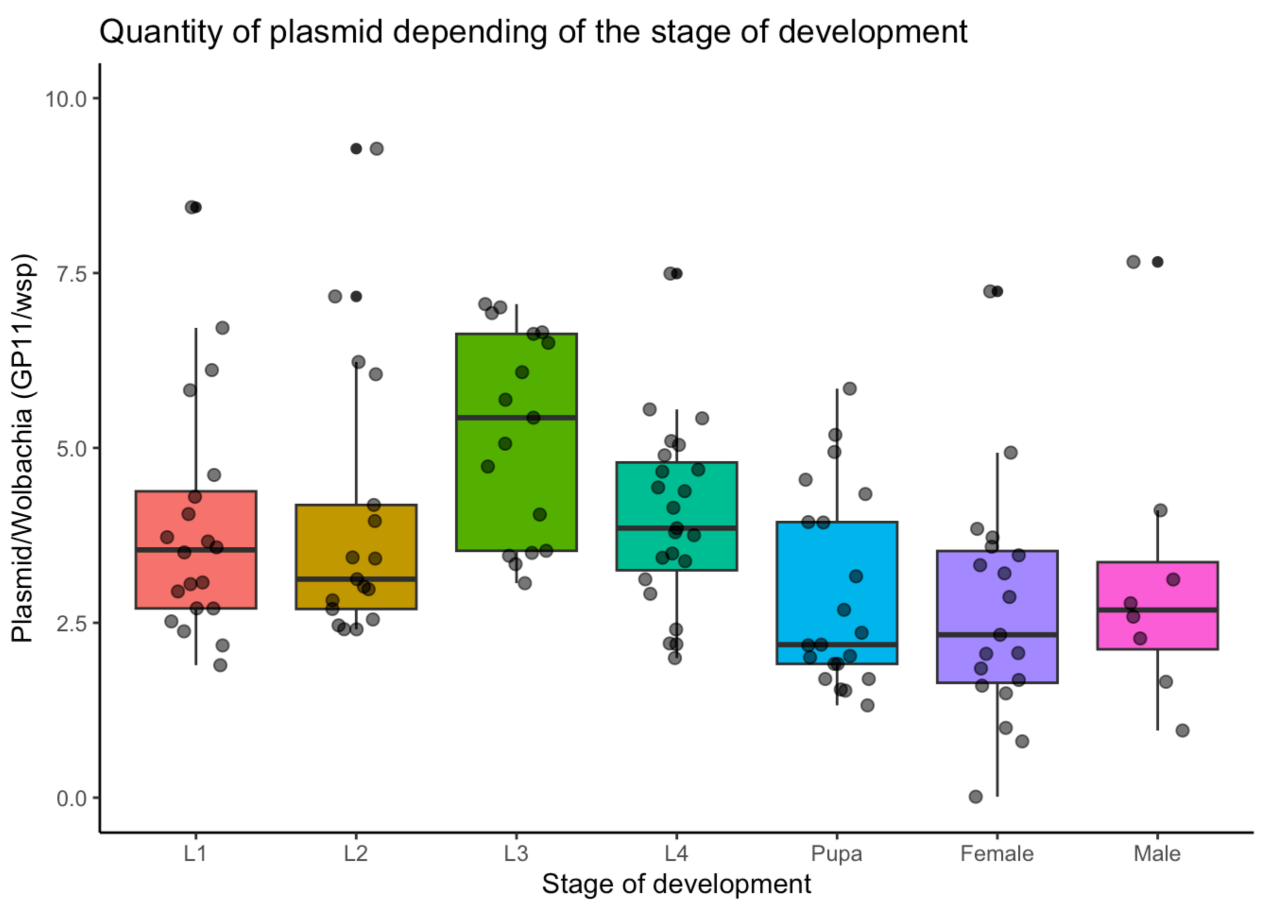

Correlation Analysis by Developmental Stage

Visualization

```r
ggplot(raw_data_mol, aes(x = wsp_ACE2, y = GP11_ACE2, color = Stade)) +
  geom_point() +
  geom_smooth(method = "lm", se = FALSE) +
  labs(title = "Ratios wsp/ACE2 vs GP11/ACE2 pour chaque stade",
       x = "wsp/ACE2",
       y = "GP11/ACE2") +
  theme_minimal()
```

```
## `geom_smooth()` using formula = 'y ~ x'
```

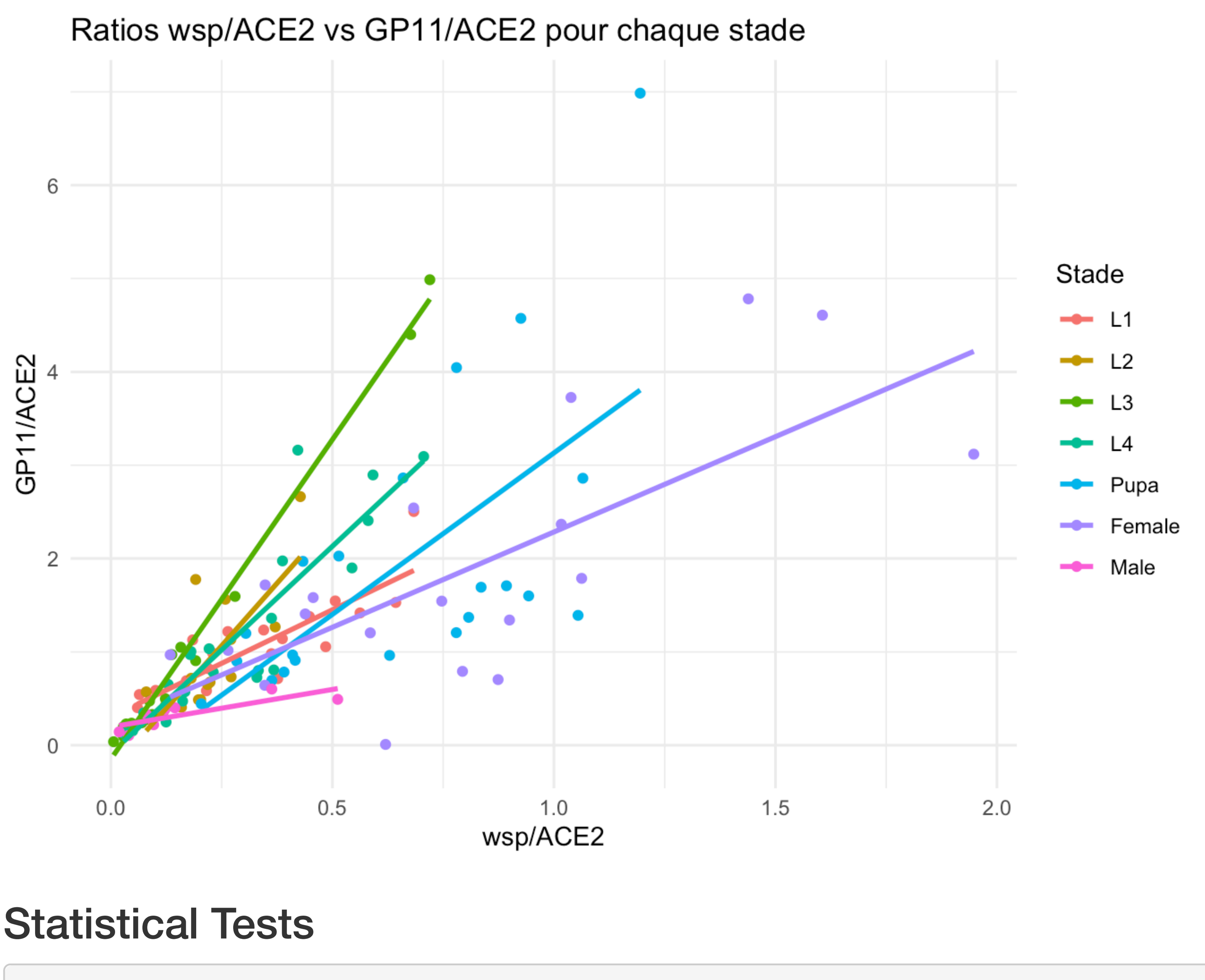

Statistical Tests

```r
# Correlation and Shapiro-Wilk tests by stage

# Step 1: Calculate correlations and p-values globally and by stage
correlation_tests <- raw_data_mol %>%
  group_by(Stade) %>%
  summarise(
    correlation = cor(wsp_ACE2, GP11_ACE2, method = "pearson"),
    p_value = cor.test(wsp_ACE2, GP11_ACE2)$p.value
  )

# Step 2: Fit linear models and extract residuals for each stage
residuals_check <- raw_data_mol %>%
  group_by(Stade) %>%
  do(model = lm(GP11_ACE2 ~ wsp_ACE2, data = .)) %>%
  mutate(residuals = list(residuals(model))) %>%
  unnest(residuals)

# Step 3: Shapiro-Wilk test for normality and conditional correlation
correlation_results <- raw_data_mol %>%
  group_by(Stade) %>%
  summarise(
    # Normality test on residuals for each stage
    shapiro_p_value = shapiro.test(residuals_check$residuals[residuals_check$Stade == unique(Stade)])$p.value,
    # Pearson or Spearman correlation based on normality
    correlation = ifelse(
      shapiro_p_value > 0.05,
      cor(wsp_ACE2, GP11_ACE2, method = "pearson"),
      cor(wsp_ACE2, GP11_ACE2, method = "spearman")
    ),
    # p-value for the appropriate correlation test
    p_value = ifelse(
      shapiro_p_value > 0.05,
      cor.test(wsp_ACE2, GP11_ACE2, method = "pearson")$p.value,
      cor.test(wsp_ACE2, GP11_ACE2, method = "spearman")$p.value
    )
  )

# Step 4: Display all results
print(correlation_tests)
print(correlation_results)
```

Comparison of Wolbachia quantities between species

```r
combined_data %>%
  group_by(Stade, Espece) %>%
  summarise(p_value = shapiro.test(wsp_ACE2)$p.value)

wilcox.test(wsp_ACE2 ~ Espece, data = combined_data[combined_data$Stade == "Femelle",])
wilcox.test(wsp_ACE2 ~ Espece, data = combined_data[combined_data$Stade == "L1",])
t.test(wsp_ACE2 ~ Espece, data = combined_data[combined_data$Stade == "L2",], var.equal = TRUE)
wilcox.test(wsp_ACE2 ~ Espece, data = combined_data[combined_data$Stade == "L3",])
t.test(wsp_ACE2 ~ Espece, data = combined_data[combined_data$Stade == "L4",], var.equal = TRUE)
wilcox.test(wsp_ACE2 ~ Espece, data = combined_data[combined_data$Stade == "Male",])
wilcox.test(wsp_ACE2 ~ Espece, data = combined_data[combined_data$Stade == "Pupa",])
```
