# Supplementary material for: Wolbachia and its pWCP plasmid show differential dynamics during the development of Culex mosquitoes
Source: Microbiol Spectr. 2025 Mar 31;13(5):e00046-25. doi: 10.1128/spectrum.00046-25 (PMC12054023; doi:10.1128/spectrum.00046-25)
Supplement: Supplemental material — Table S1; Supplemental table legends. [file spectrum.00046-25-s0002.docx]

**Supplementary Material.**

*Wolbachia* and its pWCP plasmid show differential dynamics during the development of *Culex* mosquitoes

**Supplementary Table 1: Sequences and references of primers used for qPCR analysis**

| **Target gene** | **Primer name** | **Primer sequence** | **Reference** |
| --- | --- | --- | --- |
| *ace2*  (*Culex sp.)* | Acequantidir | 5’-GCAGCACCAGTCCAAGG-3’ | (1) |
|  | Acequantirev | 5’-CTTCACGGCCGTTCAAGTAG-3’ |  |
| *wsp*  (*Wolbachia)* | Wolpipdir | 5’-AGAATTGACGGCATTGAATA-3’ | ﻿(2) |
|  | Wolpiprev | 5’-CGTCGTTTTTGTTTAGTTGTG-3’ |  |
| *GP11*  (pWCP):  5’- ttaataactacaaacactcaagcaagaaaaaggagcttttgctcgcacagaaacatcgtttttagcgtcgttcttcaaaatcgtataattatacacgttatcggggcaaggtatccctagacggtttctttcaggaaaccacatgatgaactcactgaggacatcggcgatatcatcggtgaactcagtttcagtatgaatcttttcaaactcatcaaatatcaaataatgaaaatctctttccgctttctcaagaaagatatgaccttgctcaacaaactgactgcaagactgaaacacttccatctttgacctcgttcttcttgagcctaaaatcagtgtgtctggcaatagcattcgaagatctgcgatcaccttaaagccaataccgtgttcttccacgactactgcgtgcagcctatgctcatatttactgagaaactcatgaatatcgtgttcaactgaactcgggtcaagctttcttgccaataaatccacccatactccacatctttttggctgatgggcaaaatcactcttcgtaaagaacggctgaaacactcctaccgctgttctatcaagtgttttcagcgaaccactgttgagatcgataaatgctaccagatctttttctcctattttctcataaggaaaatcaatattcctaataatgtcttgcaatactatacttagtattaacttctgtgtcat-3’ | GP11F | 5’-GCTCAACAAACTGACTGCAA-3’ | Newly designed from (3) |
|  | GP11R | 5’-TTGACCCGAGTTCAGTTGAA-3’ |  |

**Supplementary Table 2: Raw qPCR data for *Culex pipiens molestus***

**Supplementary Table 3: Raw qPCR data for *Culex quinquefasciatus***

**Supplementary Table 4: Raw *Wolbachia* and pWCP quantities for species comparison**

**Supplementary Note 1: Reproductible bioinformatic workflow (R code):** Analysis of Wolbachia and Plasmid Dynamics in Culex Mosquitoes

**References**

1. Weill M, Berticat C, Raymond M, Chevillon C. 2000. Quantitative Polymerase Chain Reaction to Estimate the Number of Amplified Esterase Genes in Insecticide-Resistant Mosquitoes. Anal Biochem 285:267–270.

2. Berticat C, Rousset F, Raymond M, Berthomieu A, Weill M. 2002. High *Wolbachia* density in insecticide–resistant mosquitoes. Proc R Soc Lond B Biol Sci 269:1413–1416.

3. Reveillaud J, Bordenstein SR, Cruaud C, Shaiber A, Esen ÖC, Weill M, Makoundou P, Lolans K, Watson AR, Rakotoarivony I, Bordenstein SR, Eren AM. 2019. The *Wolbachia* mobilome in *Culex pipiens* includes a putative plasmid. Nat Commun 10:1051.
